# High Catalytic Efficiency of a Nanosized Copper-Based Catalyst for Automotives: A Physicochemical Characterization

**DOI:** 10.3390/molecules27217402

**Published:** 2022-10-31

**Authors:** Amaia Soto Beobide, Anastasia M. Moschovi, Georgios N. Mathioudakis, Marios Kourtelesis, Zoi G. Lada, Konstantinos S. Andrikopoulos, Labrini Sygellou, Vassilios Dracopoulos, Iakovos Yakoumis, George A. Voyiatzis

**Affiliations:** 1 Foundation for Research and Technology, Institute of Chemical Engineering Sciences FORTH/ICE-HT, 26504 Patras, Greece; 2 MONOLITHOS Catalysts & Recycling Ltd., 11476 Athens, Greece; 3 Department of Physics, University of Patras, 26504 Patras, Greece

**Keywords:** copper catalyst, Ce–Zr mixed oxides, ageing, PGMs substitution, CuPdRh/CeZr washcoat, three-way catalysts, Raman, XDR, BET, SEM, XPS

## Abstract

The global trend in restrictions on pollutant emissions requires the use of catalytic converters in the automotive industry. Noble metals belonging to the platinum group metals (PGMs, platinum, palladium, and rhodium) are currently used for autocatalysts. However, recent efforts focus on the development of new catalytic converters that combine high activity and reduced cost, attracting the interest of the automotive industry. Among them, the partial substitution of PGMs by abundant non-PGMs (transition metals such as copper) seems to be a promising alternative. The PROMETHEUS catalyst (PROM100) is a polymetallic nanosized copper-based catalyst for automotives prepared by a wet impregnation method, using as a carrier an inorganic mixed oxide (CeO_2_-ZrO_2_) exhibiting elevated oxygen storage capacity. On the other hand, catalyst deactivation or ageing is defined as the process in which the structure and state of the catalyst change, leading to the loss of the catalyst’s active sites with a subsequent decrease in the catalyst’s performance, significantly affecting the emissions of the catalyst. The main scope of this research is to investigate in detail the effect of ageing on this low-cost, effective catalyst. To that end, a detailed characterization has been performed with a train of methods, such as SEM, Raman, XRD, XRF, BET and XPS, to both ceria–zirconia mixed inorganic oxide support (CZ-fresh and -aged) and to the copper-based catalyst (PROM100-fresh and -aged), revealing the impact of ageing on catalytic efficiency. It was found that ageing affects the Ce–Zr mixed oxide structure by initiating the formation of distinct ZrO_2_ and CeO_2_ structures monitored by Raman and XRD. In addition, it crucially affects the morphology of the sample by reducing the surface area by a factor of nearly two orders of magnitude and increasing particle size as indicated by BET and SEM due to sintering. Finally, the Pd concentration was found to be considerably reduced from the material’s surface as suggested by XPS data. The above-mentioned alterations observed after ageing increased the light-off temperatures by more than 175 °C, compared to the fresh sample, without affecting the overall efficiency of the catalyst for CO and CH_4_ oxidation reactions. Metal particle and CeZr carrier sintering, washcoat loss as well as partial metal encapsulation by Cu and/or CeZrO_4_ are identified as the main causes for the deactivation after hydrothermal ageing.

## 1. Introduction

It is an undeniable fact that the automotive industry plays a decisive role in our everyday life, with an economic and social impact. However, the extensive use of cars resulted in increased production of emissions [1,2,3]. In parallel, due to this phenomenon, automotive exhaust gas emission regulations have been established in order to prevent an environmental crisis [4,5]. Compliance with these regulations is among the most critical challenges faced by the automotive industry.

Automotive catalysts intensely affect these emissions in a direct way. The role of a three-way catalyst (TWC) installed on a gasoline-fueled vehicle is the simultaneous oxidation of carbon monoxide and hydrocarbons to carbon dioxide and water, as well as the reduction of nitrogen oxides to gaseous nitrogen. Any TWC consists of a catalytic powder (washcoat), deposited on the surface of a monolith substrate, which is subsequently housed in a stainless steel container using a support mat that ensures thermal and vibrational resistance [6,7]. The monolith substrate can either be ceramic, made from cordierite, or metal, and is characterized by a honeycomb structure, on the channels of which the catalytic powder is deposited (washcoated) [8]. Regarding the catalytic washcoats used in modern TWCs, they incorporate Al_2_O_3_ as a high-surface-area support and ceria or ceria-based materials (usually mixtures of CeO_2_-ZrO_2_) as carrier, due to their high oxygen storage capacity [9,10,11]. The latter property is significant for the design of a three-way catalyst, as it is required to operate efficiently under both slightly oxidative and reducing atmospheres. Taking advantage of the ability of cerium ions to easily switch between the Ce^3+^ and Ce^4+^ oxidation states, the ceria-containing material can store oxygen under oxidative conditions and also release oxygen needed for the oxidation of gases under reductive conditions [9,11]. Concerning the catalytically active phase of the washcoat material, noble metals (Pt, Pd and Rh) are currently used in autocatalysts. Specifically, Pt and/or Pd are used for catalyzing the oxidative reactions (of CO and hydrocarbons) and Rh is included for the reduction of NO_x_ to N_2_ [6,7,11,12].

An ideal catalyst should be simultaneously effective, low cost, and long lasting for its sustainability. Although both three-way catalysts (TWCs) [7,12] and diesel oxidation catalysts (DOCs) [13] are well known for their efficiency even after high mileage; additional issues have been raised, such as the stricter EU regulations for emission limits [5,14]. In addition, in spite of the fact that TWCs seem to be a more appropriate solution, they demand higher content of the catalytic phase, and therefore, higher quantities of platinum group metals (i.e., platinum, palladium and rhodium), which results in a higher capital cost for the automotive industry. In this context, research efforts have been focused on the determination of the appropriate balance between PGM loading and catalytic performance [15]. Johnson et al. [16] reported a study comparing TWC activity across a range of Pt, Pd, and Rh loadings and relative ratios. The tested catalysts were either Pd/Rh or Pt/Rh and were aged in the lab at 950 °C for 80 h (simulating the real conditions of 160,000 km for road use). As proposed, decreasing the amount of Pt or Pd (being lower than the lowest concentration of commercial catalysts) only slightly reduced the catalyst performance for HC and NOx removal. In contrast, increasing or decreasing the amount of Rh had a more significant benefit in improved catalytic activity (in terms of light-off temperature). They therefore concluded that increasing Rh levels could boost TWC performance, while Pd or Pt loadings may be decreased for cost savings, if necessary. However, in any case, the high capital cost of PGMs still remains. Thus, there is an intense need to cope with the requirement for more efficient and aging resistant catalysts.

To that end, recent efforts focus on the development of new catalytic converters that combine high activity and reduced cost (lower catalyst loading or alternative cheaper catalysts), attracting the interest of the automotive industry, bearing at the same time scientific, economic and environmental impacts [17,18]. Among them, the partial substitution of an amount of PGMs by abundant non-PGMs (transition metals) seems to be a promising alternative [19]; the substitution by copper seems to be among the best choices [20,21,22,23,24,25]. However, even though several previous research efforts focus on the replacement of noble metals with low-cost metals, the commercial exploitation of such catalysts is still limited. Recently, a novel nanosized copper-based full-scale catalyst, composed of industrially used copper, palladium and rhodium, has been developed exhibiting high activity (analogous with this of the commercial catalysts), tentatively attributed to synergistic phenomena between Cu and PGM metal nanoparticles, and stability for the abatement of CO, NO and CH_4_ [20,21,26].

However, due to the slow and expensive nature of on-road and on-engine ageing catalysts, high performance even after ageing and the development of ageing testing methods are becoming increasingly important towards the sustainability of the process. In addition, PGM resources are scarce, the demand for PGMs worldwide is growing continually, leading to the necessity of management of the waste three-way catalysts and strategies for recovery of platinum group metals from them, which adds an extra cost and process [27,28]. Catalyst ageing is the loss of catalytic activity over time, which significantly affects the emissions of the catalyst. On the other hand, catalyst deactivation is defined as the process in which the structure and state of the catalyst change, leading to loss of active sites, with a consequent reduction in its performance. The main reasons that could promote catalyst deactivation are typically divided into three categories: chemical, thermal and mechanical [29,30,31,32,33,34,35]. Catalyst deactivation is usually referred to as a complex phenomenon, due to the presence of various components in the catalytic system. Among these effects, the thermal effect is among the most common causes of car catalytic converter deactivation. This deactivation process is driven by the surface energy, which is reduced with the transport, growth and coalescence of the particles, and pore elimination, which are the reasons for noble metal agglomeration and sintering of the main supporting oxides, such as Ce/Zr mixed oxides and alumina [6,9,11,36].

The main scope of this research is to investigate in detail the effect of ageing on this novel, low-cost, effective Cu-based catalyst. The significance of Cu substitution on catalytic processes has been previously noticed in detail for different causes. For instance, when copper was introduced into the conventional bimetallic zeolite catalyst to partially substitute for zinc, the dispersion of metal increases, reducing the formation of ZnO clusters, decreasing the pore blockage, and enhancing the total pore volume of catalyst [37]. In another attempt, copper nanoparticles encapsulated on SiO_2_ evaluated as efficient non-noble metal catalysts for the vapor-phase hydrogenation of γ-valerolactone (GVL) to 2-methyltetrahydrofuran (MTHF) resulted in improved MTHF selectivity [38], while Cu contribution was extended to other areas as well, such as photocatalytic degradation of organic pollutants and copper ion-based catalysts which mimic the activity of enzymes [39,40]. In our previous publication [19], the performance of full-scale PROMETHEUS catalysts of different loadings (5 or 15 gPGMs/ft3), were compared with commercial three-way catalysts covering Euro III to Euro VIb standards. The results showed that the substitution of up to 85% of platinum group metals with Cu nanoparticles led to catalysts exhibiting high catalytic efficiencies both as fresh and aged catalysts under rich-burn and lean-burn conditions [19]. The authors observed an increase in light-off temperature by ~70 °C for the aged at 1050 °C for 4 h (10% H_2_O) PROMETHEUS catalyst (15 gPGMs/ft3 loading) com-pared to the Original Euro V/VI benchmark catalyst, even though similar maximum efficiencies were obtained for the two catalysts. It should be noted that concerning the fresh catalysts, the results showed that the PROMETHEUS full-scale catalyst presented similar or even lower light-off temperatures (specially for oxidation reactions), compared to the corresponding commercial catalysts. Thus, the present study aims at shedding light on the possible alterations of the physicochemical properties that take place upon hydrothermal ageing at 1050 °C and the way they affect the observed catalytic performance for the abatement of toxic gases. The effects are expected to be related to variations of the physicochemical properties of the washcoat, which contains the active phase for catalyzing the reactions, thus only the washcoat was used in the present study. To that end, a detailed characterization has been performed through different methods, such as scanning electron microscopy (SEM), Raman, X-ray photoelectron spectroscopy (XPS), the Brunauer–Emmett–Teller method (BET), X-ray diffraction (XRD), and X-ray fluorescence (XRF), revealing the impact of ageing on the process. Based on these findings, the underlying mechanisms are proposed.

## 2. Results and Discussion

### 2.1. Physicochemical Characterization of the Catalyst

#### 2.1.1. XRF Analysis

The metal loading of the catalytic powders was determined by XRF measurements. The results of the XRF analysis of the samples are presented in Table 1, expressed as wt.%. Concerning the metal loading of the PROM100-fresh catalyst, the Cu, Pd and Rh content of the sample was found to be close to the nominal values. On the other hand, subjecting the PROM100 sample to hydrothermal ageing at 1050 °C for 4 h caused a significant variation (decrease) in the measured values of Cu (mainly) and PGMs. Specifically, the Cu loading of the PROM100-aged catalyst was found to be 0.86 wt.%, Pd loading was 0.37 wt.% and Rh 0.06 wt.%, revealing a reduction in metal content corresponding to 39.0%, 11.9% and 14.3%, for Cu, Pd and Rh, respectively. The observed reduction can be attributed either to the decomposition of metal oxides upon heating, or to vapor phase transport of the metal oxides, eventually leading to the loss of volatile metal oxides, resulting in decreased metal content.

Martin et al. [41], have previously reported that volatilization and migration along the catalyst can take place upon ageing of commercial three-way catalysts (decreased concentration of Pd).

In addition, the observed reduction in copper content, could be also attributed to the launch and removal of small copper particles formed during fracture of large copper particles. The latter could be due to the eruption of the oxide support particles that is reported to take place during its intense sintering [42].

#### 2.1.2. Raman Characterization

**Figure 1:**
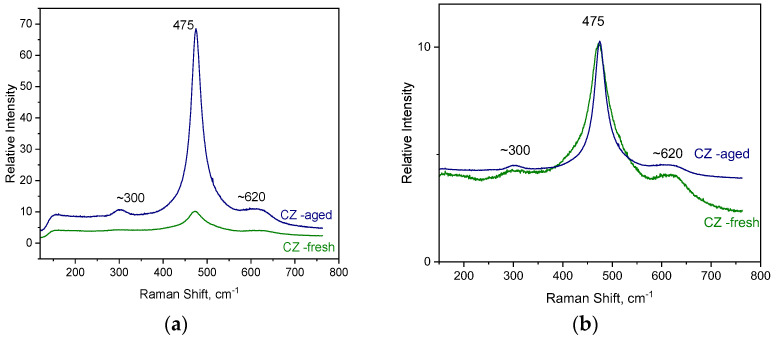
(**a**) Raman spectra of the CZ support material fresh and aged (**b**) same spectra normalized with respect to main peak.

The Raman spectrum of the CZ support material is presented in Figure 1, it presents an intense peak at 475 cm^−1^ together with broad shoulders at ~300 and 620 cm^−1^. Considering group theory, only one triply degenerated vibrational mode is Raman active for the cubic phase of CeO_2_ (the strong peak at 465 cm^−1^, F_2g_ symmetry) [43,44,45]. This band is quite intense because of high symmetry of the CeO_2_ structure and/or the high polarizability of the Ce-O bonds. On the other hand, several Raman modes are active for the tetragonal or monoclinic phase of zirconia ZrO_2_ [46,47]. In the Raman spectrum shown in Figure 1, there is no tetragonal phase from zirconia in the CZ support catalyst (Ce_0.68_Zr_0.32_O_2_) spectrum, which means that the ceria and zirconia form a solid solution and stabilize in the single cubic fluorite structure corresponding to the space group $O_{h}^{5}$ (Fm3 m). The intense peak at 475 cm^−1^ is attributed to ceria O-Ce-O stretching. In Ce_1-x_ Zr_x_O_2_ mixed oxides, by increasing the x value, there is a shift from 465 to 475 cm^−1^ due to the substitution of Ce^+4^ cations by the smaller Zr^+4^ cations [48,49]. The band at ~300 cm^−1^ denotes change in the position of the oxygen atom from their ideal fluorite structure. The broad band at 620 cm^−1^ is due to the presence of oxygen vacancy in the fluorite phase which causes defects sites in Ce–Zr oxides for the activity of the catalyst [45,50]; this band is more prominent for the fresh support. Moreover, the fact that the main peak is broader for the fresh material as it is observed in Figure 1b is an indication of smaller crystallite size in CZ-fresh material.

**Figure 2:**
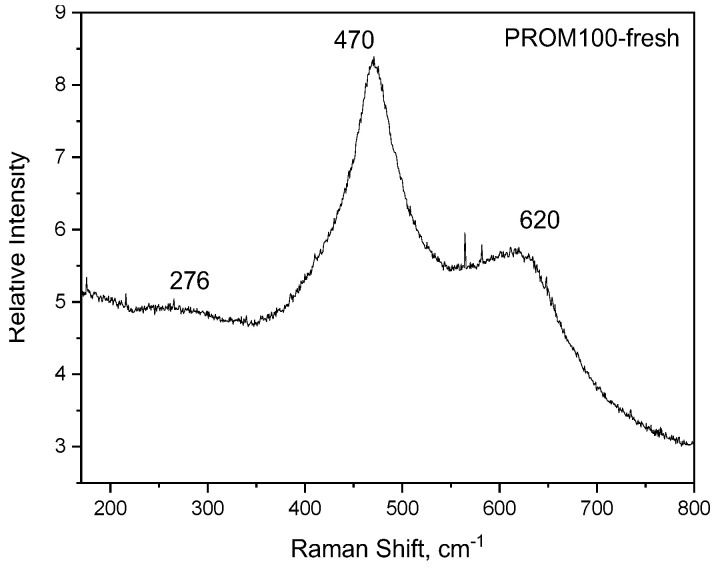
Raman spectrum of Cu/Pd/Rh doped on the CZ support, PROM100-fresh.

**Figure 3:**
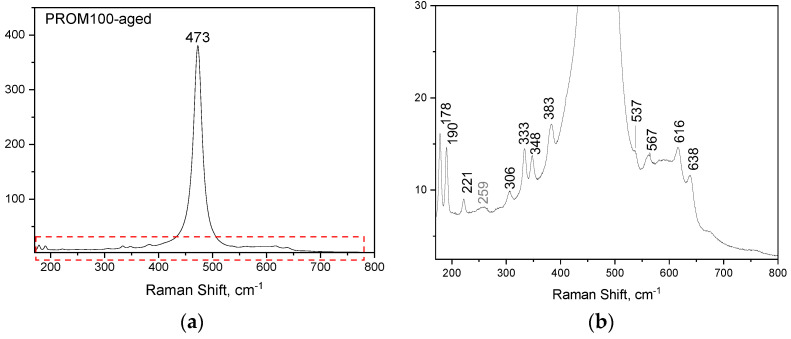
(**a**) Raman spectrum of PROM100-aged; (**b**) focus on the low-intensity bands observed.

The Raman spectrum for the PROM100-fresh and -aged samples are presented in Figure 2 and Figure 3, respectively. While the Raman spectrum of Cu/Pd/Rh doped on the CZ support-fresh (PROM100-fresh) is similar to the one corresponding to the CZ support, the Raman spectrum of PROM100-aged presents new features.

Raman spectroscopy can provide useful information about the influence of dopants on the CZ support structure and on the formation of new defective sites. Comparing the fresh samples (Figure 1a and Figure 2), the band centered at ~600 cm^−1^ and related to oxygen vacancy becomes broader and more intense for the doped catalysts (PROM100-fresh) than in the case of CZ support. Interestingly, after the inclusion of dopants to the CZ support, this peak becomes more intense, suggesting that the addition of the doping elements causes an increase in the amount of defects and in consequence more activity of the catalyst.

Regarding the aged samples, the most evident feature in the Raman spectrum, occurring due to the hydrothermal ageing process, is the increase in the intensity of the peak at ~475 cm^−1^. For the doped sample (PROM100-aged), several new spectral features appeared which are assigned to the zirconia (ZrO_2_) monoclinic phase, which is thermodynamically stable below l400 °C [47]. Moreover, the most intense Raman peak corresponding to zirconia tetragonal phase at 259 cm^−1^ is also noticeable in the Raman spectrum. The overall Raman picture suggests structural modifications upon ageing in the bulk of doped CZ mixed oxides.

#### 2.1.3. XRD Characterization

**Figure 4:**
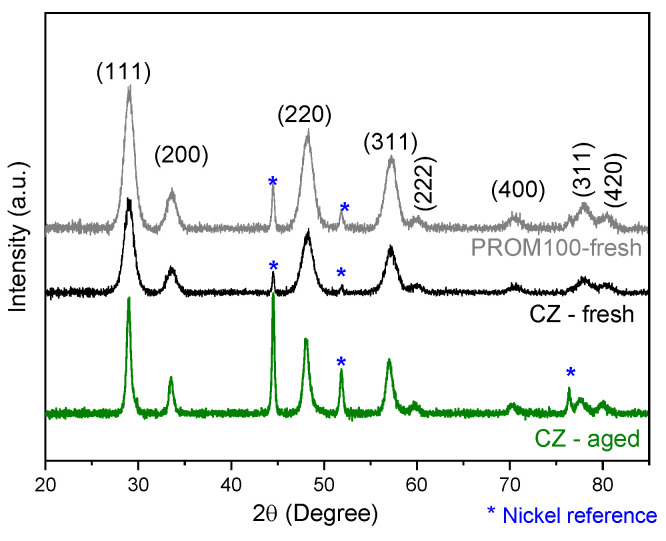
XRD patterns of the CZ- and PROM100-fresh samples. Refractive peaks indicated with asterisk correspond to Ni which was added to samples for calibration purposes.

A qualitative analysis of the diffractograms (Figure 4) showed that CZ-fresh and PROM100-fresh exhibited reflection bands appearing mainly at 29, 33.6, 48.3, 57.3, 60, 70.7, 78 and 80° 2θ angles matching the cubic fluorite structure Ce_0.75_Zr_0.25_O_2_ and space group Fm3m (JCPDS 00-028-0271). No traces of the monoclinic phase were observed. The XRD pattern for the CZ-aged samples is also depicted. The pattern revealed that the diffraction peaks narrowed with ageing treatment which is attributed to increased crystallinity and/or growth of fine grains. However, the overall position of the reflection peaks remains at the same angles indicating that the cubic crystal structure is maintained. Refractive peaks indicated with asterisks belong to external Ni that was added for calibration with 2θ = 44.5°, 51.8° and 76.4° (JCPDS 03-1051).

**Figure 5:**
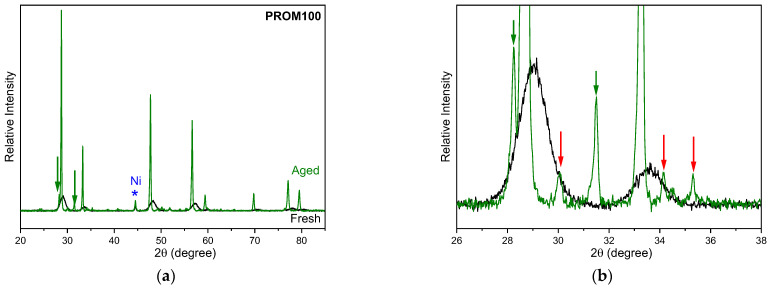
(**a**) XRD diffractograms for PROM100-fresh and PROM100-aged samples; (**b**) focus on the low-intensity peaks observed. The refractive peak indicated with asterisk correspond to Ni, which was added to samples for calibration purposes.

In Figure 5, the PROM100-aged sample is presented together with the fresh one for comparison. The intense reflection peaks belong to the cubic fluorite structure and are found to be a contribution of CeO_2_-(JCPDS 00-043-1002) and Ce_0.75_Zr_0.25_O_2_-(JCPDS 00-028-0271). Two new peaks indicated with green arrows are recognized to belong to monoclinic (P21/c) zirconia ZrO_2_ (JCPDS 00-037-1484) while very low intensity reflection peaks could be attributed to tetragonal Ce_0.1_Zr_0.9_O_2 (_JCPDS 01-088-2395) and are indicated with red arrows in Figure 5b. This is consistent with the Raman results. The PROM100-fresh sample is present in the Ce_0.75_Zr_0.25_O_2_ cubic fluorite structure and is transformed to the CeO_2_-Ce_0.75_Zr_0.25_O_2_ cubic fluorite structure, monoclinic zirconia and a very low amount of tetragonal zirconia Ce_0.1_Zr_0.9_O_2_ when hydrothermal ageing is applied. In addition, it is important to note that narrowing of XRD peaks is due to an increase in the crystallite size.

The phase separation of CeZrO_4_ mixed oxides upon thermal ageing has been previously observed by other researchers [6,51,52,53,54]. Nagai et al. [51] studied three types of CeO_2_-ZrO_2_ compounds with different oxygen storage/release capacities by XRD and XAFS. Their results suggested that ageing at temperatures higher than 1000 °C, a phase separation took place, as the CeZrO_4_ solid solution was partially divided in CeO_2_ and ZrO_2_ stable phases. Moreover, the authors reported that the increase in ageing temperature from 700 up to 1200 °C led to a gradual shortening of the Ce-O bond length, making the bond more stable, which resulted in a decrease in the ability of the oxide to release oxygen.

Thus, phase separation is highly undesirable as it may lead to serious variations in catalytic properties. It should be stressed that in the case of the present study, the carrier alone was found to be thermally stable up to 1050 °C based on Raman as well XRD results presented above, where no new peaks were observed for the carrier after ageing. The changes in the CeZrO_4_ structure, mainly phase separation, were found to be facilitated by the presence of metals. This could be related to the increased interaction of metal oxides with the carrier that probably took place upon hydrothermal treatment of the PROM100 sample, as it will be discussed in a following section. The fact that such structure variations cannot be discerned may be due to the low content of metals in the samples.

#### 2.1.4. SEM-EDX Characterization

**Figure 6:**
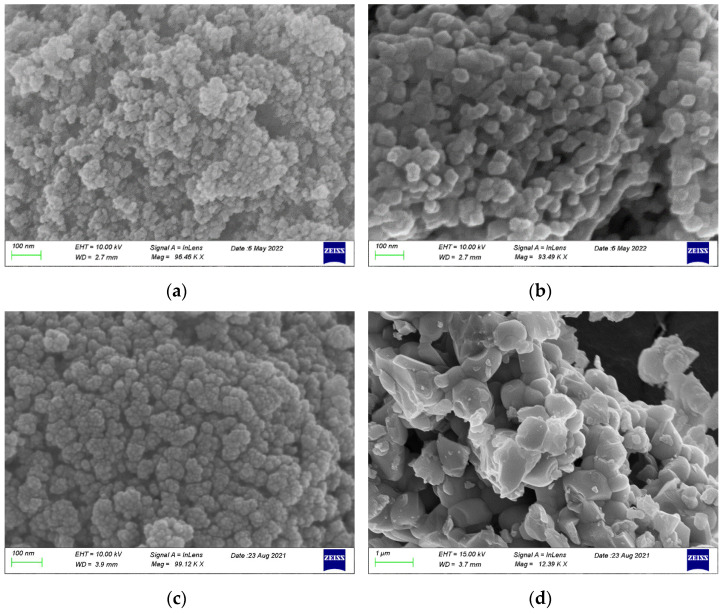
Scanning electron microscopy images of CZ-fresh (**a**) and -aged (**b**) and PROM100-fresh (**c**) and -aged (**d**).

The typical morphology of CZ support and PROM100 powders are show in Figure 6. The fresh samples exhibit mostly aggregated fine nano-sized particles. However, not obvious changes of particle size are observed suggesting that the impregnation with metals does not really alter the ceria–zirconia material. With hydrothermal ageing, the particle size increased from several nm to several tens of nm or even to the micron size in the case of metal-doped CZ (PROM100-aged). It is obvious that the sintering of the particles occurs, as evidenced by the increase in particle size of both metal particles and the particles of the support from nanometers to hundreds of nanometers and even microns.

Regarding the composition analysis from the elemental mapping in SEM-EDX (see Supplementary Data Table S1), the data are comparable to the XRF data (see Table 1).

#### 2.1.5. BET Characterization

Table 2 sums up the surface area values for samples determined by the N_2_ desorption method. The surface area of the CZ support is 91 m^2^/g and slightly decreased with metal loading to approximately 80 m^2^/g. The reduction in surface area is probably due to pore blocking of the CZ support by metals. The surface area of the aged materials (both CZ carrier alone and PROM100) showed a significant decrease in surface areas that were found to be 16 and 1 m^2^/g, in the case of CZ-aged and metal-doped PROM100-aged, respectively. The latter result is in accordance with the SEM images presented above, which revealed the sintering of the particles. Metal sintering is known to take place during the thermal treatment of three-way catalysts, leading to thermal deactivation indicated by a significant decrease in the specific surface area of the material and finally to decreased catalytic activity [35,55]. Moreover, according to Recommendation 2011/696/EU, revised on 10 June 2022, a material with a specific surface area by volume of less than 6 m^2^/cm^3^ shall not be considered as a nanomaterial. Table 1 also present the values for the specific surface area by volume. It is obvious that hydrothermal ageing of the PROM100 catalyst led to an increase in the particular size; while fresh material could be considered as a nanomaterial, the aged material does not fulfill the EU recommendations to be considered as a nanomaterial. This is in accordance with the images obtained from SEM.

#### 2.1.6. XPS Characterization

**Figure 7:**
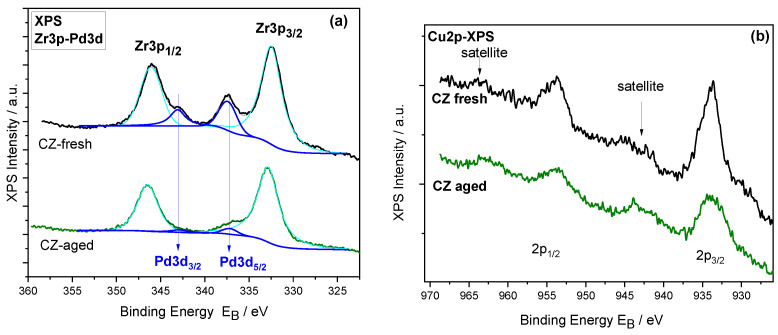
(**a**) Pd-Zr3p XP spectra window and (**b**) Cu2p XP spectra of PROM100-fresh and -aged.

**Figure 8:**
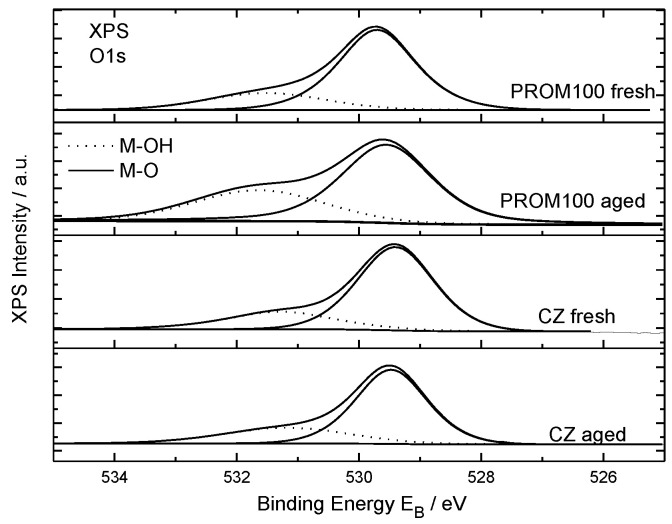
Deconvoluted O1s XP spectra of CZ carriers and PROM100-fresh and -aged.

In this work, the carriers, fresh and aged mixed cerium-zirconium oxides, and metal-doped carriers were also studied by XPS to investigate the local atomic environment of surface of materials. Rh peaks are not detected because of the nominal concentration is less than detection limit of XPS technique (approximately 0.1%at.). The Ce3d XP spectra (Figure S1 in the Supplementary Materials) are analyzed into spin-orbit doublets 3d_3/2_, 3d_5/2_, called u and v, respectively, where the satellite peaks u″, u‴, v″ and v‴ correspond to the CeO_2_ (Ce^4+^) oxidation state and the u_0_, u′, v_0_, v′ of the Ce_2_O_3_ (Ce^3+^) oxidation state of the u and v peaks. The highest binding energy peaks, u‴ and v‴ are located at 916.9 and 898.3 ± 0.1 eV, respectively [56]. From the sum of the area components of Ce^3+^ (v_0_ + v′ + u_0_ + u′) and Ce^4+^ (v + v″ + v‴ + u + u″ + u‴) the % CeO_2_ concentration is derived and shown in Table 3. The Zr3d XP spectra (Figure S2 in the Supplementary Materials) consist of a doublet with spin-orbit splitting of 2.4 eV and Zr3d_5/2_ binding energy at ~182.0 attributed to ZrO_2_ in the Ce–Zr mixed [57]. The Pd3d and Cu2p core level peak of the PROM100 catalysts are shown in Figure 7a,b, respectively. Since the Pd3d peak is overlapped with Zr3p, peak deconvolution was performed in order to separate the Pd3d component. The Pd3d_5/2_ binding energy is centered at 337.3 eV assigned to Pd oxides (Pd^2+^) [58]. The Cu2p spectra (Figure 7) consist of a doublet centered at approximately 933.5 and 953 eV corresponding to Cu2p_3/2_ and Cu2p_1/2_, respectively. It has to be mentioned that, although spectra present a low signal to noise ratio due to low Cu concentration, the Cu2p peak shape with the presence of the shake-up satellites is characteristic for Cu^+2^ (CuO) chemical state [59]. Figure 8 shows the deconvoluted O1s spectra, analyzed into to two chemical components at binding energy 529.5 ± 0.2 eV owing to lattice oxygen in the mixed oxides and to Metal-oxygen bonds (M-O, M=Pd, Cu) and to a component at higher binding energy, ~531.5 eV, denoted as O_surf_., attributed to surface adsorbed oxygen species −OH. The % ratio of the adsorbed oxygen species are shown in Table 3. The surface oxygen species are increased in the PROM catalysts because of the presence of metal atoms. From the peak intensities of Ce3d, Zr3d, O1s, Pd3d, and Cu2p divided by the relative sensitivity factors, the relative atomic compositions are derived and presented in Table 3. The atomic ratios, shown in parentheses, are close to the nominal Ce_0.68_Zr_0.32_O_2_, within experimental error. In the PROM100-fresh samples, the Cu:Pd atomic ratio is shown and is higher than the nominal (Cu/Pd = 21/7 = 3), indicating that on the surface, because Pd is incorporated into the bulk of the catalyst. This is more pronounced in the aged sample, where Pd is almost eliminated from the surface.

If we compare the data between the surface technique (XPS) with the bulk techniques (EDX and XRF), we observe that the Pd is reduced on the surface but remains constant in the bulk as we move from the fresh to the aged samples. This indicates that the Pd is incorporated into the catalyst particle in the aged samples due to a sintering effect.

### 2.2. Catalytic Activity Tests

**Figure 9:**
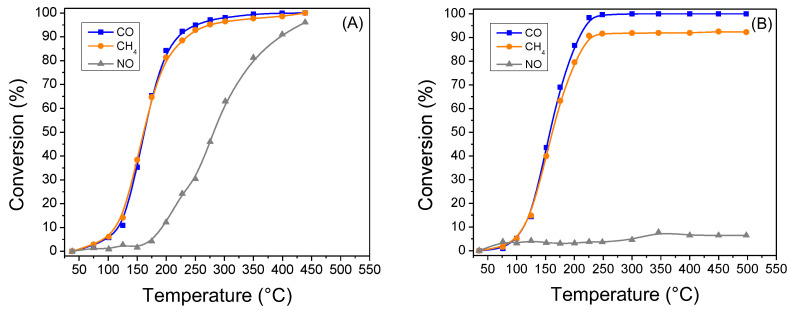
Light-off curves of the conversion of CO, CH_4_ and NO of PROM100-fresh catalytic washcoat: (**A**) under rich-burn conditions (λ = 0.99) and (**B**) under lean-burn conditions (λ = 1.03).

The synthesized catalytic powders (fresh and aged samples) were tested for their efficiency in CO, CH_4_ and NO abatement reactions on a lab-scale synthetic gas bench (SGB). The CO, CH_4_ and NO conversion curves of PROM100-fresh nanocatalytic powder as a function of temperature are presented in Figure 9 for the tests performed under both rich- and lean-burn conditions. Moreover, Table 4 summarizes the values of T_50_ and overall conversion for every reaction under investigation. Under a slightly reductive atmosphere (λ ≈ 0.99), the fresh catalyst was found to be highly active for the reactions investigated; namely CO and CH_4_ oxidation and the NO reduction reaction. Specifically, the PROM100-fresh sample was found to be active for CO abatement at temperatures higher than 75 °C, reaching full conversion at 330 °C. The same trend was observed for CH_4_ oxidation reaction, indicating the high activity of the catalyst for both oxidation reactions; this, specifies the promotional effect of Cu presence on the catalyst, which is known to favor the oxidation of CO and CH_4_ [60,61,62], additionally to Pd. The catalyst was also found to be active for the NO reduction reaction although at slightly higher temperatures (T > 175 °C), with the conversion of NO reaching a maximum value of 96% at 440 °C.

The experiment performed over the PROM100-fresh catalyst under lean-burn conditions (Figure 9B), revealed a similar performance regarding the CO oxidation reaction, while CH_4_ oxidation was found to be limited to 93% at temperatures higher than 450 °C. The most significant difference observed was related to the catalyst’s performance for the NO reduction reaction, as the PROM100-fresh catalyst exhibited NO conversions lower than 8% for the whole temperature range investigated under oxidative conditions (λ ≈ 1.03). The latter may be attributed to the surface oxidation of Rh nanoparticles of the catalyst.

**Figure 10:**
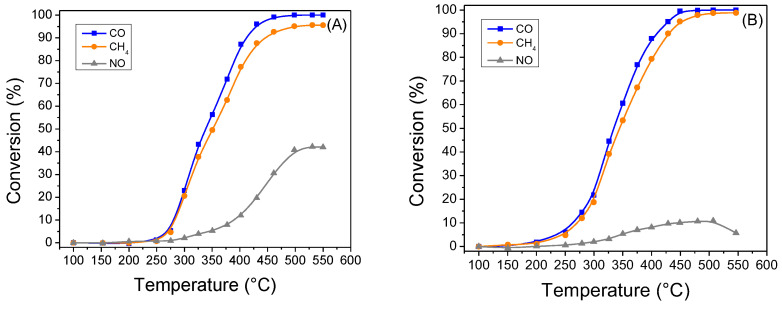
Light-off curves of the conversion of CO, CH_4_ and NO of PROM100-aged catalytic washcoat: (**A**) under rich-burn conditions (λ = 0.99) and (**B**) under lean-burn conditions (λ = 1.03).

Concerning the performance of the PROM100-aged catalyst, Figure 10 includes the variation of CO, CH_4_ and NO conversion as a function of reaction temperature. It can be easily observed that the hydrothermal ageing of the catalyst led to a shift of the catalytic activity at significantly higher temperatures in all cases. This could be attributed to the metal sintering as well as the oxidation of metal nanoparticles upon hydrothermal ageing. Specifically, for CO oxidation reaction under rich-burn conditions, the PROM100-aged catalyst seems to be active at temperatures higher than 250 °C, reaching full CO conversion at T > 460 °C. A similar trend is observed for CH_4_ oxidation reaction, although the conversion of CH_4_ is limited to 96%. On the other hand, the catalyst exhibits significantly different performance compared to the fresh sample regarding the NO reduction reaction. Specifically, the maximum NO conversion was found to be 42% at temperatures higher than 500 °C. The latter is probably related to the effect of ageing on Rh nanoparticles, which are probably transformed to a Rh_2_O_3_ form which is not active for the NO reduction reaction. For the experiment performed under slightly lean-burn conditions, a similar performance is observed for the PROM100-aged catalyst, with the catalyst being active for oxidation reactions at temperatures higher than 200 °C (significantly higher than those obtained for the fresh sample), while the catalyst exhibited low activity for NO abatement (maximum NO conversion was 11%).

Table 4 includes the values of T_50_ and overall conversion for every reaction under investigation presented above. It can be seen that under rich-burn conditions, the T_50_ for CO and CH_4_ oxidation reactions are shifted to higher temperatures by 175 and 190 °C, respectively for the case of the aged sample compared to the fresh one. On the other hand, the hydrothermal ageing resulted in a catalyst with decreased activity for performing the reduction of NO. Similarly, under lean-burn conditions, the T_50_ for CO and CH_4_ oxidation reactions in the case of the aged PROM100 sample were shifted to higher temperatures by 174 and 186 °C, respectively, with respect to the fresh sample. Both the fresh and hydrothermally aged samples presented low conversion values for the NO reduction reaction.

It should be stressed that the results of the present study can be regarded as indicative of what should take place over a full-scale TWC, where the washcoat is deposited on the walls of the honeycomb ceramic monolith. In that case, the washcoat accounts for a small percentage of the total weight of the sample (usually 10–20 wt.%), so the observed variations in physicochemical properties as well as catalytic activity would be significantly less important. Moreover, the presence of Al_2_O_3_ in the TWC formulation, which is characterized by high thermal stability and high specific surface area, increases the resistance of the metal phase and the CeZrO_4_ carrier to sintering [6].

Concerning the high activity of the PROM100-fresh sample under both rich- and lean-burn conditions, this has been previously ascribed to synergistic phenomena between Cu and PGM metal nanoparticles and the ceramic support (material with oxygen storage capacity) at low temperatures [21].

Upon thermal ageing, the PROM100 catalytic washcoat was found to have different physicochemical properties (BET surface area, surface structure, surface concentration and metal loading). The variation of such properties after ageing led to lead to a catalyst that partly retained its activity towards CO and HC oxidation reactions (although the light-off temperatures were shifted to higher values), but its efficiency for NO conversion was significantly lowered. There are several studies that have investigated the effect of thermal ageing on catalyst activity for TWCs, using either commercial TWCs (including cordierite), washcoat powders or model PGM containing catalysts [9,33,34,41,42,53,63,64,65]. In most of these studies, active metal sintering has been described as the main cause of catalyst degradation. However, other causes have also been identified as responsible for the observed decrease in catalytic performance after thermal ageing, including sintering of the oxygen storage material and the alumina, migration of PGMs into the carrier lattice, encapsulation of PGMs, loss of interaction between PGMs and support as well as volatilization and washcoat loss.

It should be noted that all the aforementioned effects of thermal ageing on catalytic performance found in literature refer to the performance of noble metals (Pt or Pd and Rh) and their interaction with the support material under ageing conditions. Since the PROMETHEUS catalyst is the first catalyst characterized by the substitution of part of PGMs with Cu, the effect of thermal ageing on the local structure of Cu nanoparticles is expected to have an important role in the observed degradation of catalytic activity after ageing. Thus, in an attempt to explain the observed catalytic performance of the PROM100 sample before and after ageing, as presented in Figure 7 and Figure 8, some of the causes presented above can be adopted, based also on the results of physicochemical characterization. The results of BET measurements as well as Raman and XRD indicated the increase in CeZrO_4_ crystallite size after the hydrothermal treatment at 1050 °C. The results clearly indicate that sintering of the support took place after thermal treatment. Moreover, such high temperatures favor active metal (Cu, Pd and Rh) sintering blocking pores of the support reducing further the BET surface area of the metal containing samples compared to the carrier alone (Table 2). Several studies have shown that the decrease in catalytic performance in three-way catalysts can be related to the degradation of oxygen storage material (CeZrO_4_) due to its decreased activity for the Ce^4+^ to Ce^3+^ redox reaction [9,33,51,53,64]. The latter has been previously attributed to the gradual shortening of the Ce-O bond length with increasing ageing temperature, making difficult the release of oxygen [9,51]. Moreover, the metal sintering contributes to a decrease in the contact between active metals and the oxide support, reducing the total three-phase boundary (gas-metal-oxide) length, and thus the total active surface which is exposed to the reactants [33,34,53].

XPS results showed that the relative concentration of Cu oxide on the surface increased for the aged sample (mainly in the Cu^2+^ oxidation state). Similar observations have been reported previously for monometallic supported Cu catalysts [66,67]. Wang et al. [66], recently studied the effect of thermal ageing (calcination temperatures of 300–500 °C) on the formation and nature of active sites on a series of Cu-CeO_2_ nanorods catalysts with varying Cu content, for CO oxidation reaction. They reported the surface enrichment of Cu atoms during thermal treatment at 800 °C, which they attributed to the segregation of CuO_x_ onto the ceria surface due to the decomposition of oxides such as the bulk Cu_y_Ce_1-y_O_2-x_ solid solution which was formed at lower temperatures but was unstable at this temperature. They concluded that in the catalyst calcined at 800 °C, CuO_x_ species supported on a Cu-doped CeO_2_ thin film layer were formed, leading to enhanced activity for CO oxidation. In another study, Yoshida et al. [67], investigated a model Cu catalyst supported on Al_2_O_3_, thermally aged at different temperatures up to 1000 °C (in the presence of 10% H_2_O in air) for its activity for stoichiometric NO-CO-C_3_H_6_-O_2_ reaction. They reported that the ageing of the catalyst at 900 °C led to improved NO reduction efficiency at high temperatures (X_NO_ = 70% at 600 °C). However, a further increase in ageing temperature at 1000 °C significantly decreased oxidation and reduction efficiencies due to the structural changes in Cu/Al_2_O_3_—namely formation of highly crystalline CuAl_2_O_4_ and α-Al_2_O_3_. At the same time, they reported that the surface concentration of Cu increased with increasing aging temperature (as evidenced by XPS).

Concerning Pd species evolution after ageing, the XPS results showed the Pd oxide relative concentration on the surface was decreased after thermal treatment at 1050 °C. Moreover, XPS results suggest that Pd is found on the surface in the form of PdO. One possible explanation could involve the encapsulation of Pd by CuO or CeZrO_2_. The encapsulation of PGMs at high temperatures has been previously reported in studies investigating the thermal deactivation of TWCs [42,64,68,69,70]. In this respect, the minimization of surface energy has been identified as the main driving force for metal encapsulation by support [42]. The sintering of the support results in an expansion of lattice parameters, introducing a compressive stress on the upper layer of CeZrO_4_ surface. Thus, oxide cations and anions can diffuse along the CeZrO_4_ surface towards the metal particles, where the stress could be released by the eruption of the support, leading to partial encapsulation of the metal [42].

Several literature studies have reported that under oxidizing conditions, similar to those applied for the ageing of the PROM100 catalyst in the present study, the PdO is resistant to sintering [34,42,65], contrary to Rh_2_O_3_ [9]. The latter could explain the reduced ability of PROM100-aged to convert NO even under rich-burn conditions. Several authors concluded that with increasing temperature under oxidizing conditions, the interaction between Rh-oxide and the support is becoming stronger, leading to the diffusion of Rh_2_O_3_ into the support and further increase in Rh particles [9,65].

Taking into account the aforementioned literature findings in combination with the characterization results, the reasons behind the observed decrease in catalytic efficiency of the PROM100 catalyst after ageing may be explained by a combination of metal phase and CeZr carrier sintering, volatilization, washcoat loss as well as partial metal encapsulation. The sintering of the metals and the CeZrO_4_ carrier is evidenced by the significant decrease in the surface area of both the carrier and the metal-doped catalyst after hydrothermal ageing which is also expected to have a detrimental effect on the oxygen storage capacity properties of the carrier. Moreover, the sintering of the metals and the carrier is expected to cause variations on the interactions between Cu, PGMs and the carrier which have been named as the main reason for the observed activity of the fresh catalyst. The partial encapsulation of Cu and PGMs either by the support or other metals found in higher quantities cannot be ruled out, even though no direct indications were provided by the physicochemical characterization results, probably due to the low metal content of the PROM100 sample. Volatilization as well as washcoat loss have also been confirmed by the combination of XRF analysis and XPS measurements of the PROM100-fresh and -aged samples. Upon hydrothermal treatment at 1050 °C, the oxide characterized by the highest vapor pressure (Cu oxide) is moving towards the surface, leading to increased Cu surface concentration as evidenced by XPS and subsequently, a fraction of volatile Cu oxide is partly evaporated through vapor-phase transport, interpreting the decreased Cu content measured by XRF.

In summary, the most significant outcomes obtained from the results of the various characterization techniques are gathered in Table 5. These results are correlated with the recorded catalytic activity performance in the aged samples. A general observation is that even if a sintering effect is noticed through the characterization of samples after ageing, the catalyst remains active. It is noteworthy though that catalytic performance is a multifunctional parameter, and all aspects should be considered (Table 4).

## 3. Materials and Methods

### 3.1. Catalyst Preparation

The catalyst powder was prepared using commercially available chemical reagents, without further purification. The following chemical reagents were used: ceria–zirconia mixed inorganic oxide, Ce_0.68_Zr_0.32_O_2_ (CZ, Wanfeng Technology, Shaoxing, China) and ammonium hydroxide solution (Merck, percent concentration 25 wt.%). Copper (II) nitrate trihydrate (Acros Organic, purity 99%—solid form), palladium (II) nitrate solution (Heraeus, solution assay 17.94 wt.%) and rhodium (III) nitrate solution (Hereaus, solution assay 9.27 wt.%) were used as metal precursors. The heterogeneous PROMETHEUS (PROM100) catalyst was synthesized by the conventional wet impregnation method following a procedure that has been previously described [20]. Mass calculations of materials used were performed, in order to achieve the desired metal loading of 2 wt.%, at a molar ratio of Cu/Pd/Rh = 21/7/1. After the synthesis and the calcination of the catalytic powder at 500 °C, for 1 h, the catalytic washcoat prepared (PROM100-fresh) was sieved at <125 μm, obtaining a fine granulometry, in order to improve the specific surface area required for further processing. A fraction of the synthesized PROM100 catalyst was subjected to catalyst ageing following a certified protocol. According to the protocol, each sample was heated up to 1050 °C for 4 h under 10% H_2_O air flow. A high-temperature calcination furnace was used equipped with an atmospheric air electric pump for air introduction to the heating chamber. Upstream of the furnace, the air stream was passing through a thermostated water saturator operating at 46 °C, for the introduction of 10% H_2_O in air mixture into the furnace chamber.

Samples PROM100-fresh and PROM100-aged consist of catalyst washcoat (Cu/Pd/Rh doped on the CZ support)—no cordierite included. It should be noted that for the preparation of a real-scale three-way catalyst, the catalytic powder should be washcoated on the walls of a ceramic cordierite-based ((Mg, Fe)_2_Al_4_Si_5_O_18_) monolith. However, the aim of the present article is the investigation of the effect of hydrothermal ageing on the physicochemical properties and the catalytic efficiency of the washcoat alone, which is the active phase responsible for catalyzing the reactions. On the other hand, the presence of the cordierite would be expected to cause less variation on the physicochemical properties and performance of the catalyst. Thus, throughout the text, the term “fresh” refers to a catalyst that has not been used at all, while the term “aged” refers to a catalyst that has been artificially aged by submitting it to the above-mentioned hydrothermal ageing protocol. This specific technique is well documented in the literature and EU Directives for simulating more than 60,000 km of mileage of a car.

In summary, the samples under study are the ceria–zirconia mixed oxide support (CZ-fresh and -aged) and the PROMETHEUS catalyst washcoat consisting of the CZ support doped with metals Cu/Pd/Rh (PROM100-fresh and -aged).

### 3.2. Materials Characterization

The metal loading of the bulk materials was determined by performing X-ray fluorescence analysis of the synthesized fresh and aged powders using a Vanta Olympus (2017, Waltman, MA, USA) XRF analyzer, specially calibrated for the precise measurement of each metal as described elsewhere [20]. Prior to the analysis, the catalytic powders (granulometry below 125 μm) were dried at 120 °C for 2 h and then the XRF samples were prepared for analysis by pressing ~5 g of the powder inside polyethylene cups. The analysis was performed by measuring 10 repeated scans (1 scan/90 s).

Raman spectroscopy (a non-destructive chemical analysis technique) was used to obtain detailed information about the chemical structure, phase and polymorphism and molecular interactions of the materials. Raman spectra were recorded on a T-64000 (Jobin Yvon-Horiba) micro-Raman system utilized in a single-spectrograph configuration. The excitation wavelength (514.5 nm) was provided by a DPSS laser (Cobolt Fandango TMISO laser). Dispersion and detection of the Raman photons were performed by an 1800 grooves/mm grating and a Spectraview-2DTM liquid N2-cooled CCD detector, respectively. The laser was focused on the samples by a 50× microscope objective with a power of 0.5 mW on sample. The resolution was kept constant in all experiments at ~2.5 cm^−1^.

X-ray diffraction (XRD) spectra were recorded for the structural characterization of the CZ support and PROM100 samples by using a Bruker D8 Advance diffractometer equipped by a Cu lamp (λCuKa = 1.54046 Å) at a scan speed 2 sec/step over a range 20–80° (2θ). A certain amount of external Ni sample was added to the samples under study (CZ-fresh and -aged and PROM100-fresh and -aged) for calibration (Ni 2θ = 44.5°, 51.8° and 76.4° JCPDS 03-1051).

Scanning electron microscope (SEM) images were obtained using a Zeiss SUPRA 35VP-FEG instrument, operating at 5–20 keV. The system is equipped with EDX (Bruker GmbH, Quanta 200) and BSE (K E Developments), which enabled elemental analyses.

The determination of the specific surface area (SBET) of the samples was achieved via N_2_ sorption isotherms at −196 °C, using a Quantachrome Autosorb IQ-C-MP apparatus. The samples were outgassed at 120 °C for 2 h.

X-ray photoelectron spectroscopy (XPS) analysis was performed in an Ultra-High-Vacuum (UHV) system equipped with SPECS Phoibos 100-1D-DLD hemispherical electron analyzer and a non-monochromatized dual-anode Mg/Al x-ray source and operating at a pressure of 10^−9^ mbar. The photoelectron spectra were recorded using an Mg-Kα (hν = 1253.6 eV) non-monochromatized source (300 W) and an analyzer pass energy of 10 eV giving a Full Width at Half Maximum (FWHM) of 0.85 eV for Ag3d_5/2_ line. The acquisition and fitting were realized with the commercial software SpecsLab Prodigy (Specs GmbH, Berlin, Germany). The atomic ratios were calculated from the intensity (peak area) of the XPS peaks weighted with the corresponding relative sensitivity factors (RSFs). The analyzed area was 7 × 10 mm^2^

### 3.3. Catalytic Activity Measurements

The lab-scale testing of the synthesized catalytic powders (both as fresh and aged samples) was performed in an in-house synthetic gas bench (SGB) setup. The gas feed was prepared using gas cylinders containing known quantities of mixtures to acquire a gas composition simulating the real working conditions of a petrol engine’s exhaust system. The gaseous species (CO, CH_4_, NO, CO_2_, and O_2_, all diluted in N_2_ used as balance gas) are controlled using mass flow controllers. It should be noted that a separate N_2_ flow passes through a thermostated H_2_O saturator in order to facilitate the introduction of steam to the gas mixture (saturator temperature: 65 °C). After mixing, the final gas stream was fed in the fixed-bed U-type quartz reactor which contains the catalytic powder. The reactor is placed inside a glass tube electrical furnace, where the temperature is controlled and monitored with a K-type thermocouple, while the effluent gas is analyzed continuously using two multi-gas analyzers (GA-200 PVT, HNL Ltd., Mumbai, India) connected in series downstream the reactor. The analysis system used enables the simultaneous analysis of CO_2_, CO, O_2_, NO, NO_2_ and CH_4_. It should be noted that several different hydrocarbon species can be found in a real exhaust gas, having different chemical activity in contact with the catalyst. In the present study, CH_4_ was used for the preparation of the simulated gasoline exhaust mixture, as the main species of total hydrocarbon emissions of vehicles [71].

In a typical experiment, 100 mg of the catalyst powder (<125 μm) was placed in the reactor and then the gas mixture was prepared. Two different feed compositions were used for the experiments to simulate the different conditions of a petrol-fueled vehicle exhaust, corresponding to slightly lean-burn (λ ≈ 1.03) and slightly rich-burn (λ ≈ 0.99) conditions. The lambda ratio (λ) is an expression of the air to fuel ratio of a mixture, divided by the stoichiometric air to fuel ratio. Lambda values higher than 1 correspond to oxidative conditions, while λ < 1 corresponds to a slightly reductive atmosphere. The gas feed consisted of 1% CO, 12% CO_2_, 800 ppm NO, 2500 ppm CH_4_, 0.91 or 0.95% O_2_ and 10% H_2_O (in N_2_ balance). The different λ values were obtained by varying the flow of the reactants in the feed gas maintaining a total gas flow of 300 cm^3^/min in all tests. After collecting the baseline values of the reactants by-passing the reactor, the gas stream was introduced in the reactor and the concentrations of reactants and products were recorded following a step-wise increase in temperature in order to obtain the light-off temperatures and conversions efficiency of the samples for CO and CH_4_ oxidation reactions as well as the NO reduction reaction. The conversion of reactants (CO, CH_4_ and NO) is calculated using Equation (1):

(1) $$X_{i}=\frac{C_{in}-C_{out}}{C_{in}}\cdot 100\%$$

where *X_i_* is the conversion (%) of gas *i*, *C_in_* the inlet concentration (%) of gas *i* and *C_out_* the outlet concentration (%) of gas *i*.

## 4. Conclusions

In this study, the effect of ageing on a low-cost novel trimetallic copper-based nano-catalyst comprising copper, palladium and rhodium supported on a Ce_0.68_Zr_0.32_O_2_ carrier (CZ) was investigated. To that end, a detailed characterization has been performed through different methods. The addition of metal dopants causes several changes in the CZ support structure which have been noticed by Raman spectroscopy and XRD in excellent agreement between the two techniques.

Ageing of the PROM100 catalyst at 1050 °C for 4 h (with 10% H_2_O in air) increased the light-off temperatures by more than 175 °C, compared to the fresh sample, without affecting the overall efficiency of the catalyst for CO and CH_4_ oxidation reactions which reached 100% and >93%, respectively, under both rich- and lean-burn conditions. Rh sintering and/or encapsulation led to decreased catalytic activity towards the NO reduction reaction under rich-burn conditions. On the other hand, both PROM100-fresh and -aged catalysts showed low conversion values for the NO reduction reaction due to the low Rh content of the washcoat. Metal particle and CeZr carrier sintering, volatilization, washcoat loss as well as partial metal encapsulation by Cu and/or CeZrO_4_ are identified as the main causes for the deactivation after hydrothermal ageing.

## Figures and Tables

**Table 1:** Measured and theoretically expected copper and PGM loading of the synthesized powders (fresh and aged samples).

	Expected (wt.%)			Expected (wt.%)		
Samples	Cu	Pd	Rh	Cu	Pd	Rh
PROM100-fresh	1.41	0.42	0.07	1.45	0.48	0.07
PROM100 CZ-aged	0.86	0.37	0.06			

**Table 2:** Textural properties of the CZ support and the PROM100 catalyst.

Sample	Surface Area m^2^/g	Volume-Specific Surface Area ^1^ m^2^/cm^3^
CZ-fresh	91	594
CZ-aged	16	104
PROM100-fresh	81	528
PROM100-aged	1	6

^1^ Density (CeZrO_4_) = 6.52 g/cm^3^.

**Table 3:** XPS results of CZ and PROM100-fresh and -aged materials.

	% Atomic Concentration (±0.5) (Atomic Ratio)						% at. Ce^4+^	% at. O_surf_./O
Samples	Ce	Zr	O	Pd	Cu	Cu:Pd		
CZ-fresh	23.3 (0.70)	12.3 (0.37)	64.4 (1.93)	-	-		76.8%	23.5
CZ-aged	28.0 (0.84)	11.5 (0.35)	60.5 (1.82)	-	-		73.7%	26.7
PROM100 CZ-fresh	23.7 (0.76)	9.3 (0.30)	60.5 (1.94)	1.16	5.5	4.7	86.4%	28.2
PROM100 CZ-aged	17.7 (0.57)	10.0 (0.32)	65.8 (2.11)	0.17	6.4	37.6	84.7%	30.8

**Table 4:** T50 and maximum efficiency values for the abatement of CO, CH4 and NO, obtained for the tested catalysts.

Sample	Rich-Burn Conditions (λ ≈ 0.99)						Lean-Burn Conditions (λ ≈ 1.03)					
	CO Oxidation		CH_4_ Oxidation		NO Reduction		CO Oxidation		CH_4_ Oxidation		NO Reduction	
	T_50_ (°C)	Maximum Efficiency (%)	T_50_ (°C)	Maximum Efficiency (%)	T_50_ (°C)	Maximum Efficiency (%)	T_50_ (°C)	Maximum Efficiency (%)	T_50_ (°C)	Maximum Efficiency (%)	T_50_ (°C)	Maximum Efficiency (%)
PROM100-fresh	162	100	161	100	282	96	158	100	162	93	-	8
PROM100-aged	338	100	351	96	-	42	334	100	344	99	-	11

**Table 5:** Highlights from the different characterization methods used in this study and correlation with the catalytic activity.

	Technique						
Sample	XRF	Raman	XRD	SEM	SBET	XPS	Cata. Activity * Max. Efficiency (λ ≈ 0.99)
**CZ-fresh**		Ce_1-x_ Zr_x_O_2_	Ce_0.75_Zr_0.25_O_2_	Aggregates of nano-particles (<100 nm)		Ce_0.70_Zr_0.37_O_1.93_	
**CZ-aged**		Ce_1-x_ Zr_x_O_2_	Ce_0.75_Zr_0.25_O_2_ (narrow peaks)	Bigger particles (micro size)	No-nano	Ce_0.84_Zr_0.35_O_1.82_	
**PROM100-fresh**	Cu: 1.41% Pd: 0.42%	Ce_1-x_ Zr_x_O_2_	Ce_0.75_Zr_0.25_O_2_ (broad peaks)	Aggregates of nano-particles (<100 nm)		Ce_0.76_Zr_0.30_O_1.94_ Cu:Pd = 4.7	CO and CH_4_: 100% NO: 96%
**PROM100-aged**	Cu: 0.86% Pd: 0.37%	-high increase in Ce-O bonds -presence ZrO_2_ monoclinic -contribution ZrO_2_ tetragonal	-CeO_2_ -ZrO_2_ monoclinic and tetragonal (narrow peaks)	Bigger particles (micro size)	No-nano	Ce_0.57_Zr_0.32_O_2.1_ Cu:Pd = 37.6	CO and CH_4_: ~100% NO:42%

* Refer to Table 4.

## Data Availability

The raw data will be available from corresponding author upon reasonable request.

## Supplementary Materials

The following supporting information can be downloaded at: https://www.mdpi.com/article/10.3390/molecules27217402/s1, Table S1: Measured copper and palladium loading from SEM-EDX mapping analysis of the PROM100 fresh and aged samples; Figure S1: Deconvoluted Ce3d XP Spectra of CZ support material and PROM100 fresh and aged catalysts and Figure S2: Zr3d XP Spectra of CZ support material and PROM100 fresh and aged catalysts.
