# Supplementary material for: High Catalytic Efficiency of a Nanosized Copper-Based Catalyst for Automotives: A Physicochemical Characterization
Source: Molecules. 2022 Oct 31;27(21):7402. doi: 10.3390/molecules27217402 (PMC9657973; doi:10.3390/molecules27217402)
Supplement: Supplementary file 1 [file molecules-27-07402-s001.zip › molecules-1973485-supplementary.pdf]

Supplementary Material

High catalytic efficiency of nanosized copper-based catalyst for automotive: A physicochemical characterization

Amaia Soto Beobide [1,*], Anastasia M. Moschovi [2], Georgios N. Mathioudakis [1], Marios Kourtelesis [2], Zoi G. Lada [1], Konstantinos S. Andrikopoulos [1,3], Labrini Sygellou [1], Vassilios Dracopoulos [1], Iakovos Yakoumis [2,*] and George A. Voyiatzis [1]

Table S1: Measured copper and palladium loading from SEM-EDX mapping analysis of the PROM100 fresh and aged samples.

% w concentration (±1.5%)			
**Samples**	**Cu (±0.2%)**	**Pd (±0.1%)**	**Cu:Pd**
PROM100-fresh	1.44	0.38	3.8
PROM100-aged	0.51	0.32	1.6

Ce3d and Zr3d XPS spectra of the fresh and aged CZ support and PROM100 catalysts

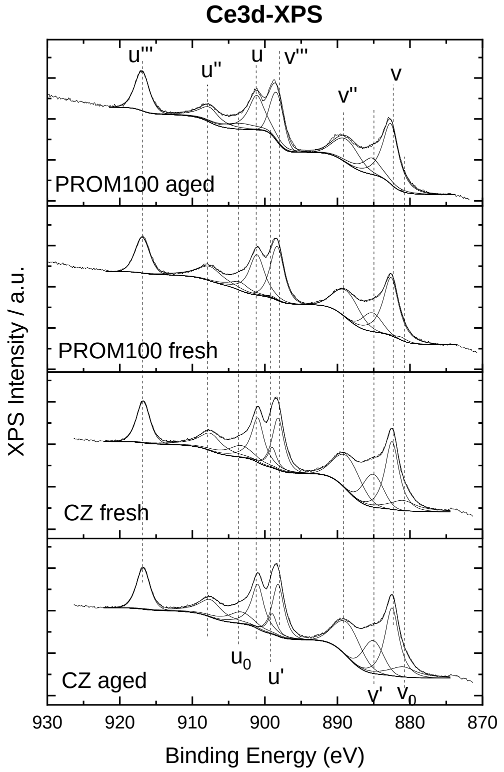

Figure S1: Deconvoluted Ce3d XP Spectra of CZ support material and PROM100 fresh and aged catalysts.

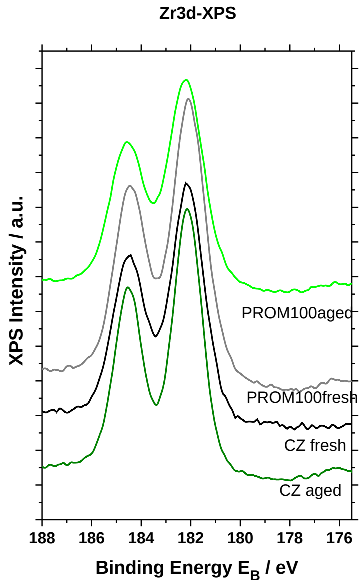

Figure S2: Zr3d XP Spectra of CZ support material and PROM100 fresh and aged catalysts.
